# Exploring Disparities in Pancreatic Ductal Adenocarcinoma Outcomes among Asian and Pacific Islander Subgroups

**DOI:** 10.1158/2767-9764.CRC-24-0177

**Published:** 2024-08-19

**Authors:** Christopher Wu, M. Chandler McLeod, Zhixing Song, Herbert Chen, John Bart Rose, Smita Bhatia, Andrea Gillis

**Affiliations:** 1 General Surgery, University of Alabama at Birmingham, Birmingham, Alabama.; 2 Pediatric Hematology/Oncology, University of Alabama at Birmingham, Birmingham, Alabama.

## Abstract

Pancreatic ductal adenocarcinoma (PDAC) is a challenging malignancy with known disparities in outcomes across ethnicities. Studies specifically investigating PDAC in Asian populations are sparse, overlooking the rich diversity within this group. This research seeks to fill that gap by examining survival differences across the broad spectrum of Asian ethnicities, acknowledging the complexity and varied experiences within these communities. Utilizing the National Cancer Database from 2004 to 2019, we categorized patients into East Asian, Southeast Asian, South Asian, and Pacific Islander groups. Non-Asians or Pacific Islanders were excluded. Overall survival was analyzed using a Cox hazards model. The study consisted of 13,254 patients. Most patients were East Asian (59.4%, *n* = 7,866). Southeast Asians exhibited the poorest survival in unadjusted analysis (HR, 1.32; 95% confidence interval, 1.23–1.42; *P* < 0.001) compared with South Asians who exhibited the best survival. Multivariable analysis revealed significantly worse survival for East Asians and Pacific Islanders relative to South Asians, whereas Southeast Asians’ results were not significantly different. Asian subgroup differences notably affect PDAC outcomes. Research on genetic and cultural aspects, especially in Southeast Asians, and tackling health disparities are crucial for enhancing survival in this diverse disease.

**Significance:**

This study highlights the significant survival disparities among Asian subgroups with pancreatic cancer, utilizing a large national database. By differentiating among East Asian, Southeast Asian, South Asian, and Pacific Islander groups, it underscores the need for tailored research and healthcare approaches. Addressing these differences is essential for developing culturally sensitive interventions and potentially improving outcomes in a disease that uniquely affects these diverse populations.

## Introduction

Pancreatic ductal adenocarcinoma (PDAC), notorious for its insidious onset and aggressive course, continues to challenge clinicians and researchers alike (1). PDAC has already eclipsed breast cancer, emerging as the third foremost cause of cancer-related mortality in the United States, as evidenced by recent epidemiologic data. This shift in ranking raises heightened concern given the anticipated trajectory; projections indicate that PDAC is poised to be the number one cause of cancer mortality by 2040 (2, 3). As research into the etiology and treatment strategies of this malignancy progresses, the impact of ethnicity on disease outcomes has garnered increased attention (4). For example, minority races and ethnicities such as African Americans and Asians are reported to have inferior outcomes when compared with their White counterparts (5, 6). However, it is crucial to acknowledge that the term “Asian” comprises a myriad of distinct ethnicities, each characterized by unique genetic profiles, cultural practices, level of acculturation, and environmental exposures (7).

Asia, the largest continent globally, encompasses a remarkable array of ethnicities across its diverse regions, including (roughly) East Asia, Southeast Asia, South Asia, Central Asia, and West Asia (8). From the Chinese in the East to the Punjabi in the West, the Malay in the South, and the Kazakh in the North, the sheer diversity within the Asian demographic is unparalleled (9). In the United States, each subgroup possesses its own trends of genetic predispositions, dietary habits, lifestyle choices, and access to healthcare systems, collectively contributing to a mosaic of factors that undoubtedly influence the presentation and outcomes of diseases such as PDAC.

Despite this diversity, much of the existing literature homogenizes data on PDAC among Asians, obscuring critical differences among various subgroups and limiting insightful conclusions. This study looks to resolve this limitation by systematically exploring the available evidence within the context of diverse Asian and Pacific Islander populations and examining the particularities of their subgroups, including their social determinants of health (SDOH) and access to treatment. SDOH involve a range of factors, such as economic stability, education level, social and community context, healthcare access, and neighborhood environments, which can profoundly influence health outcomes (10). By dissecting these elements, our goal is to unravel the complex intricacies that contribute to the disparities observed in PDAC outcomes. With this study, we aim to contribute to targeted research and clinical interventions that could improve outcomes for specific Asian subgroups affected by PDAC. This study is significant as previous research has established the impact of SDOH on health outcomes broadly, yet there is a notable gap in understanding how these determinants intersect specifically with racial and ethnic identities (11). By addressing this intersectionality, we aim to fill a crucial gap in the current understanding, thus enhancing the effectiveness of healthcare strategies tailored for these populations.

## Materials and Methods

### Data source

We utilized the National Cancer Database (NCDB), a collaborative initiative between the Commission on Cancer of the American College of Surgeons and the American Cancer Society. The NCDB comprehensively records 70% or more of newly diagnosed malignancies in the United States on an annual basis. Due to the utilization of a de-identified file from the NCDB, this study was deemed exempt from institutional review board approval.

### Study population

This investigation included individuals aged 18 years or older and diagnosed with PDAC within the time frame spanning from 2005 to 2019 (to allow at least 1 year of follow-up). PDAC diagnosis was identified through the International Classification of Diseases for Oncology, Third Edition histology codes: 8010, 8020, 8021, 8050, 8140 to 8145, 8190, 8200, 8210, 8211, 8255, 8260 to 8263, 8290, 8323, 8430, 8453, 8480, 8481, 8490, 8500, 8503, 8504, 8510, 8521, 8523, 8560, 8570, 8572, and 8575. Patients who were Black, White, Hispanic, American Indian, Aleutian, Eskimo, other, or unknown were excluded to focus on Asian and Pacific Islander subgroups only. Similarly, individuals diagnosed with American Joint Committee on Cancer (AJCC) stage 0 disease (*in situ*) were not included in the study population. Records for individuals with 0 days of survival from diagnosis (*n* = 2) or with surgery start recorded as occurring after the last contact (*n* = 96) were excluded. Patients were split into four groups: East Asian, Southeast Asian, South Asian, and Pacific Islander based on nationality. The East Asian cohort included Chinese, Japanese, and Korean individuals. The Southeast Asian cohort comprised Filipino, Vietnamese, Laotian, Hmong, Kampuchean (including Khmer and Cambodian), and Thai individuals. The South Asian cohort consisted of Asian Indian and/or Pakistani individuals. Finally, the Pacific Islander cohort encompassed Hawaiian, Micronesian, Chamorran, Guamanian, Polynesian, Tahitian, Samoan, Tongan, Melanesian, Fiji Islander, New Guinean, and Pacific Islander individuals. These groupings were adopted because of the classification methodology used by the Pew Research Center (12).

### Statistical analysis

We used descriptive statistics to characterize the cohort with counts and percentages used for categorical variables and means and SDs and/or quartiles used for continuous variables, as appropriate. Comparison of group characteristics was performed using *χ*^2^ tests and ANOVA for categorical and continuous variables, respectively. The primary endpoint in this present investigation was overall survival (OS) of study participants and was computed from the date of diagnosis to the date of death. Patients who were lost to follow-up were censored at the last known date of contact or the date of the last recorded medical visit before the loss of contact. Survival was initially evaluated for Asian subgroups using Kaplan–Meier methods with comparisons between groups conducted using log-rank statistics. An extended Cox model was fit to determine the association of Asian subgroups with survival while adjusting for pertinent factors including receipt of surgery as a time-dependent covariate along with other factors including demographics (age, gender, rural–urban residence, distance to hospital, N stage, M stage, AJCC staging, and diagnosis era), SDOH (insurance status, zip code area, median household income, and percent with no high school education), comorbidities [Charlson–Deyo comorbidity index (CDCI)], and treatment center characteristics (hospital region and community or academic). To assess the potential collinearity between socioeconomic status and education within our statistical models, we used generalized variance inflation factors [GVIF^(1/(2 × Df))^]. Statistical analysis was performed in R (version 4.2.1, 2022) using the tidyverse package (v2.0.0) for data cleaning and the survival package (v3.5-7) for survival analysis. Collinearity was assessed for model parameters using the vif function in the car package. All statistical tests were two-sided with statistical significance defined by *P* value < 0.05.

### Data availability

The data analyzed in this study were obtained from the NCDB at https://www.facs.org/quality-programs/cancer/ncdb.

## Results

### Demographics

The study population consisted of a total of 13,254 patients. The majority of participants were East Asian, comprising 59.3% (*n* = 7,866) of the cohort (Table 1). This was followed by Southeast Asian (22.1%, *n* = 2,933), South Asian (12.4%, *n* = 1,638), and Pacific Islander (6.2%, *n* = 817). The mean age of the cohort was 68.1 (SD, 12.7) years. Most participants were female (51.0%, *n* = 6,759). A predominant number of patients were beneficiaries of Medicare (48.5%), and most patients were treated in an academic healthcare facility (65.2%).

**Table 1:** Demographic characteristics of Asian subpopulations in patients with PDAC

Characteristic	South Asian (*N* = 1,638)	East Asian (*N* = 7,866)	Pacific Islander (*N* = 817)	Southeast Asian (*N* = 2,933)	Total (*N* = 13,254)	*P* value^a^
Age						<0.001 (1)
Mean (SD)	65.6 (12.4)	69.2 (12.6)	63.7 (13.1)	67.8 (12.3)	68.1 (12.7)	
Age group						<0.001 (2)
≥65	920 (56.2%)	5,222 (66.4%)	422 (51.7%)	1,843 (62.8%)	8,407 (63.4%)	
<65	718 (43.8%)	2,644 (33.6%)	395 (48.3%)	1,090 (37.2%)	4,847 (36.6%)	
Observation time						<0.001 (1)
Mean (SD)	21.4 (28.4)	18.1 (26.1)	19.5 (28.6)	17.0 (25.6)	18.3 (26.5)	
Time from diagnosis to treatment						0.06 (1)
Median (Q1, Q3)	22.0 (11.0, 36.0)	24.0 (11.0, 40.0)	25.0 (10.3, 44.0)	24.0 (10.0, 42.0)	24.0 (10.0, 40.0)	
Gender						<0.001 (2)
Male	922 (56.3%)	3,784 (48.1%)	404 (49.4%)	1,385 (47.2%)	6,495 (49.0%)	
Female	716 (43.7%)	4,082 (51.9%)	413 (50.6%)	1,548 (52.8%)	6,759 (51.0%)	
Insurance status						<0.001 (2)
Not insured	100 (6.1%)	259 (3.3%)	36 (4.4%)	112 (3.8%)	507 (3.8%)	
Insurance status unknown	40 (2.4%)	171 (2.2%)	10 (1.2%)	31 (1.1%)	252 (1.9%)	
Medicaid/other government programs	220 (13.4%)	745 (9.5%)	96 (11.8%)	392 (13.4%)	1,453 (11.0%)	
Medicare	646 (39.4%)	4,045 (51.4%)	358 (43.8%)	1,377 (46.9%)	6,426 (48.5%)	
Private insurance/managed care	632 (38.6%)	2,646 (33.6%)	317 (38.8%)	1,021 (34.8%)	4,616 (34.8%)	
Median household income						<0.001 (2)
<$40,227	98 (6.6%)	599 (8.4%)	38 (5.3%)	223 (8.6%)	958 (8.0%)	
$40,227–$50,353	173 (11.6%)	815 (11.4%)	99 (13.8%)	339 (13.1%)	1,426 (11.9%)	
$50,354–$63,332	268 (18.0%)	1,386 (19.3%)	191 (26.6%)	690 (26.7%)	2,535 (21.2%)	
$63,333+	949 (63.8%)	4,370 (60.9%)	390 (54.3%)	1,330 (51.5%)	7,039 (58.9%)	
Percent with no high school degree						<0.001 (2)
<6.3%	507 (34.1%)	2,236 (31.2%)	158 (22.0%)	419 (16.2%)	3,320 (27.8%)	
6.3%–10.8%	410 (27.6%)	1,992 (27.8%)	255 (35.5%)	663 (25.7%)	3,320 (27.8%)	
10.9%–17.5%	263 (17.7%)	1,270 (17.7%)	162 (22.6%)	605 (23.4%)	2,300 (19.2%)	
17.6%+	308 (20.7%)	1,673 (23.3%)	143 (19.9%)	895 (34.7%)	3,019 (25.2%)	
Rural–urban residence						<0.001 (2)
Urban	33 (2.1%)	192 (2.5%)	85 (10.7%)	76 (2.6%)	386 (3.0%)	
Metropolitan	1,533 (97.6%)	7,402 (97.0%)	708 (88.9%)	2,788 (97.1%)	12,431 (96.6%)	
Rural	4 (0.3%)	38 (0.5%)	3 (0.4%)	6 (0.2%)	51 (0.4%)	
Greater circle distance						<0.001 (2)
Short (<12.5 minutes)	952 (63.8%)	5,274 (73.3%)	410 (56.6%)	1,878 (72.2%)	8,514 (70.8%)	
Intermediate (12.5–50 minutes)	444 (29.7%)	1,458 (20.3%)	196 (27.0%)	556 (21.4%)	2,654 (22.1%)	
Long (>50 minutes)	97 (6.5%)	467 (6.5%)	119 (16.4%)	167 (6.4%)	850 (7.1%)	
Charlson–Deyo score						<0.001 (2)
0	1,036 (63.2%)	5,515 (70.1%)	484 (59.2%)	1,928 (65.7%)	8,963 (67.6%)	
1	475 (29.0%)	1,736 (22.1%)	231 (28.3%)	723 (24.7%)	3,165 (23.9%)	
2	76 (4.6%)	357 (4.5%)	54 (6.6%)	178 (6.1%)	665 (5.0%)	
3+	51 (3.1%)	258 (3.3%)	48 (5.9%)	104 (3.5%)	461 (3.5%)	
Diagnosis era						<0.001 (2)
2005–2009	305 (18.6%)	1,626 (20.7%)	143 (17.5%)	674 (23.0%)	2,748 (20.7%)	
2010–2014	504 (30.8%)	2,536 (32.2%)	256 (31.3%)	962 (32.8%)	4,258 (32.1%)	
2015–2019	829 (50.6%)	3,704 (47.1%)	418 (51.2%)	1,297 (44.2%)	6,248 (47.1%)	
T stage						<0.001 (2)
T1	109 (7.0%)	566 (7.7%)	65 (8.6%)	218 (7.9%)	958 (7.7%)	
T2	409 (26.1%)	1,739 (23.6%)	213 (28.3%)	722 (26.3%)	3,083 (24.8%)	
T3	391 (25.0%)	1,978 (26.8%)	180 (23.9%)	626 (22.8%)	3,175 (25.5%)	
T4/Tx	658 (42.0%)	3,084 (41.9%)	295 (39.2%)	1,182 (43.0%)	5,219 (42.0%)	
N stage						<0.001 (2)
N0	828 (53.2%)	4,245 (57.9%)	477 (63.9%)	1,532 (56.0%)	7,082 (57.3%)	
N1	440 (28.3%)	1,712 (23.4%)	151 (20.2%)	659 (24.1%)	2,962 (23.9%)	
N2/Nx	288 (18.5%)	1,372 (18.7%)	119 (15.9%)	545 (19.9%)	2,324 (18.8%)	
M stage						0.003 (2)
M0	841 (53.9%)	4,042 (55.1%)	426 (56.1%)	1,385 (51.0%)	6,694 (54.1%)	
M1/Mx	719 (46.1%)	3,296 (44.9%)	333 (43.9%)	1,333 (49.0%)	5,681 (45.9%)	
AJCC staging						0.005 (2)
Stage I	197 (12.9%)	1,061 (14.7%)	118 (15.8%)	387 (14.4%)	1,763 (14.4%)	
Stage II	401 (26.2%)	1,846 (25.5%)	187 (25.0%)	590 (21.9%)	3,024 (24.8%)	
Stage III	203 (13.3%)	980 (13.5%)	106 (14.2%)	357 (13.3%)	1,646 (13.5%)	
Stage IV	729 (47.6%)	3,354 (46.3%)	337 (45.1%)	1,359 (50.5%)	5,779 (47.3%)	
Facility location						<0.001 (2)
West South Central	120 (7.6%)	445 (5.8%)	34 (4.4%)	128 (4.5%)	727 (5.6%)	
East North Central	225 (14.2%)	504 (6.5%)	34 (4.4%)	195 (6.8%)	958 (7.4%)	
East South Central	33 (2.1%)	76 (1.0%)	11 (1.4%)	16 (0.6%)	136 (1.0%)	
Middle Atlantic	555 (35.0%)	1,435 (18.6%)	38 (4.9%)	246 (8.6%)	2,274 (17.5%)	
Mountain	26 (1.6%)	202 (2.6%)	37 (4.8%)	75 (2.6%)	340 (2.6%)	
New England	54 (3.4%)	231 (3.0%)	10 (1.3%)	89 (3.1%)	384 (3.0%)	
Pacific	275 (17.3%)	3,808 (49.3%)	543 (70.4%)	1,780 (61.9%)	6,406 (49.4%)	
South Atlantic	263 (16.6%)	854 (11.0%)	50 (6.5%)	222 (7.7%)	1,389 (10.7%)	
West North Central	36 (2.3%)	174 (2.3%)	14 (1.8%)	123 (4.3%)	347 (2.7%)	
Facility type						<0.001 (2)
Community	466 (29.4%)	2,638 (34.1%)	249 (32.3%)	1,154 (40.2%)	4,507 (34.8%)	
Academic	1,121 (70.6%)	5,091 (65.9%)	522 (67.7%)	1,720 (59.8%)	8,454 (65.2%)	
Type of surgical diagnostic procedure						0.005 (2)
None	544 (33.2%)	2,943 (37.5%)	308 (37.7%)	1,066 (36.4%)	4,861 (36.7%)	
Biopsy	998 (61.0%)	4,473 (57.0%)	459 (56.2%)	1,661 (56.7%)	7,591 (57.3%)	
Surgery	95 (5.8%)	438 (5.6%)	50 (6.1%)	205 (7.0%)	788 (6.0%)	
Palliative care						<0.001 (2)
No	1,450 (87.5%)	6,910 (87.1%)	658 (79.9%)	2,607 (87.8%)	11,625 (86.8%)	
Yes	207 (12.5%)	1,027 (12.9%)	166 (20.1%)	362 (12.2%)	1762 (13.2%)	
Vital status						<0.001 (2)
Alive	510 (30.8%)	1,866 (23.4%)	176 (21.3%)	632 (21.3%)	3,184 (23.7%)	
Dead	1,147 (69.2%)	6,105 (76.6%)	649 (78.7%)	2,337 (78.7%)	10,238 (76.3%)	
Surgery						<0.001 (2)
No	1,199 (73.2%)	5,850 (74.4%)	597 (73.1%)	2,288 (78.0%)	9,934 (75.0%)	
Yes	421 (25.7%)	1,893 (24.1%)	212 (25.9%)	629 (21.4%)	3,155 (23.8%)	
Unknown	18 (1.1%)	123 (1.6%)	8 (1.0%)	16 (0.5%)	165 (1.2%)	
Chemotherapy						<0.001 (2)
No	988 (60.3%)	5,229 (66.5%)	551 (67.4%)	2,073 (70.7%)	8,841 (66.7%)	
Yes	631 (38.5%)	2,534 (32.2%)	254 (31.1%)	842 (28.7%)	4,261 (32.1%)	
Unknown	19 (1.2%)	103 (1.3%)	12 (1.5%)	18 (0.6%)	152 (1.1%)	
Radiation						0.001 (2)
No	1,320 (80.6%)	6,464 (82.2%)	668 (81.8%)	2,482 (84.6%)	10,934 (82.5%)	
Yes	267 (16.3%)	1,107 (14.1%)	127 (15.5%)	373 (12.7%)	1,874 (14.1%)	
Unknown	51 (3.1%)	295 (3.8%)	22 (2.7%)	78 (2.7%)	446 (3.4%)	

Linear model ANOVA and Pearson *χ*^2^ test.

a *P* values set for significance <0.05.

The mean follow-up time was 18.3 (SD: 26.5) months. Southeast Asians had the shortest mean follow-up time of 17.0 (25.6) months, with East Asians, Pacific Islanders, and South Asians followed for 18.1 (26.1), 19.5 (28.6), and 21.4 (28.4) months on average, respectively. Most patients (67.6%, *n* = 8,963) presented with no significant comorbid conditions (CDCI = 0). East Asians had the largest proportion of patients with a CDCI score of 0 (70.3%, *n* = 5,515), whereas Pacific Islanders had the smallest (59.2%, *n* = 484).

In examining the socioeconomic data of the cohort based on SDOH, it was observed that a predominant fraction of the patients (58.9%, *n* = 7,039) lived in zip codes belonging to the uppermost income quartile, with an income threshold (> $63,333). Disparities were apparent across ethnic subgroups; Southeast Asians presented the lowest representation within this income quartile (>$63,333) at 51.5% (*n* = 1,330), whereas South Asian participants had the highest representation at 63.8% (*n* = 949). Analysis of educational attainment at the zip code level revealed distinct patterns across ethnic groups. Notably, within the Southeast Asian patient group, a considerable portion, amounting to 34.7% (*n* = 895), was in the quartile reflecting the lowest educational attainment (17.6%+ with no high school degree). This incidence was markedly higher than the next highest group (East Asian) at 23.3% (*n* = 1,673), indicating a notable divergence in educational backgrounds within this subset of the patient population.

Few patients in the cohort (23.8% *n* = 3,155) underwent surgery. Southeast Asians were least likely to undergo surgical procedures, with only 21.4% (*n* = 629) receiving surgery. Chemotherapy and radiation utilization was similarly low across the cohort, with only 32.1% (*n* = 4,261) receiving chemotherapy and 14.1% (*n* = 1,874) receiving radiation. Interestingly, Southeast Asians also had the lowest proportion of patients receiving chemotherapy and radiation at 28.7% (*n* = 842) and 12.7% (*n* = 373), respectively.

### OS

**Figure 1:**
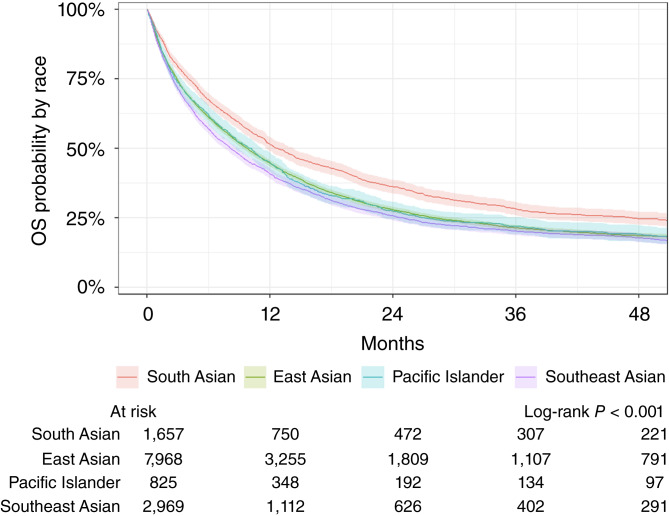
Kaplan–Meier survival curves for the Asian subgroup analysis.

In the Kaplan–Meier analysis of 4-year OS rates stratified by ethnicity, estimates indicated that South Asian patients experienced the highest survival at 24.4% [95% confidence interval (CI), 22.2–26.9]. This was followed by Pacific Islanders with an OS of 19.2% (95% CI, 16.5–22.4), East Asians with an OS of 18.3% (17.4–19.3), and Southeast Asians with the worst 4-year survival at 17.5% (95% CI, 16.0–19.1; log-rank *P* < 0.001; Fig. 1). In an unadjusted Cox model analysis, the lower survival for Southeast Asians corresponded to a HR 1.32 times higher (95% CI, 1.23–1.42; *P* < 0.001) than that of South Asians (Table 2). Furthermore, with their intermediate survival, Pacific Islanders and East Asians both had higher HRs than South Asians (Pacific Islander HR, 1.21; 95% CI, 1.10–1.33; *P* < 0.001; East Asian HR, 1.22; 95% CI, 1.15–1.30; *P* < 0.001).

**Table 2:** Univariable survival analysis for patients diagnosed with PDAC

Characteristic	HR	95% CI	*P* value^a^
Asian subgroup			
South Asian	—	—	
East Asian	1.22	1.15–1.30	<0.001
Pacific Islander	1.21	1.10–1.33	<0.001
Southeast Asian	1.32	1.23–1.42	<0.001
Age group			
≥65	—	—	
<65	0.58	0.55–0.60	<0.001
Gender			
Male	—	—	
Female	1	0.96–1.04	>0.9
Insurance status			
Not insured	—	—	
Insurance status unknown	0.93	0.78–1.11	0.4
Medicaid/other government programs	0.85	0.75–0.96	0.008
Medicare	1.18	1.06–1.32	0.003
Private insurance/managed care	0.8	0.72–0.89	<0.001
Median household income			
<$40,227	—	—	
$40,227–$50,353	1.02	0.93–1.12	0.7
$50,354–$63,332	0.98	0.90–1.06	0.6
$63,333+	0.95	0.88–1.03	0.2
No high school degree			
<6.3%	—	—	
6.3%–10.8%	1.08	1.03–1.15	0.004
10.9%–17.5%	1.11	1.04–1.18	<0.001
17.6%+	1.11	1.05–1.17	<0.001
Rural–urban residence			
Urban	—	—	
Metropolitan	0.85	0.76–0.95	0.005
Rural	0.95	0.69–1.31	0.8
Greater circle distance			
Short (<12.5 minutes)	—	—	
Intermediate (12.5–50 minutes)	0.75	0.72–0.79	<0.001
Long (>50 minutes)	0.7	0.65–0.76	<0.001
Facility location			
West South Central	—	—	
East North Central	1.03	0.92–1.15	0.6
East South Central	1.22	0.99–1.51	0.06
Middle Atlantic	0.91	0.82–1.00	0.055
Mountain	1.27	1.09–1.47	0.002
New England	1.04	0.89–1.20	0.6
Pacific	1.30	1.19–1.43	<0.001
South Atlantic	1.03	0.93–1.15	0.5
West North Central	1.12	0.96–1.30	0.14
Facility type			
Community	—	—	
Academic	0.75	0.72–0.78	<0.001
Charlson–Deyo score			
0	—	—	
1	1.07	1.02–1.12	0.005
2	1.23	1.13–1.34	<0.001
3+	1.57	1.41–1.74	<0.001
Diagnosis era			
2005–2009	—	—	
2010–2014	0.8	0.76–0.84	<0.001
2015–2019	0.65	0.62–0.69	<0.001
T stage			
T1	—	—	
T2	2.07	1.88–2.29	<0.001
T3	2.53	2.30–2.79	<0.001
T4/Tx	2.79	2.54–3.07	<0.001
N stage			
N0	—	—	
N1	1.51	1.44–1.59	<0.001
N2/Nx	1.55	1.47–1.63	<0.001
M stage			
M0	—	—	
M1/Mx	2.37	2.28–2.47	<0.001
AJCC staging			
Stage I	—	—	
Stage II	1.75	1.62–1.90	<0.001
Stage III	2.53	2.32–2.76	<0.001
Stage IV	4.50	4.18–4.85	<0.001
Surgery (time-dependent)			
No			
Yes	0.30	0.29–0.32	<0.001

a *P* values set for significance <0.05.

### SDOH and survival

On multivariable analysis, survival outcomes were associated with educational level. Specifically, reduced survival was observed with decreasing rates of educational attainment. Compared with patients living in zip codes with the highest educational attainment, those in zip codes with the next highest educational attainment had an HR of 1.08 (95% CI, 1.03–1.15; *P* = 0.004) and those in zip codes with the lowest educational attainment (10.9%–17.5% or >17.5%) both had an HR of 1.11 (95% CIs, 1.04–1.18 and 1.05–1.17, respectively; both *P* < 0.001).

When subsetting for each race, the intersection between SDOH and race did not demonstrate a linear relationship across different racial groups. For Southeast Asians, no significant associations were found between educational attainment, income, or rural–urban residency (*P* > 0.05). In contrast, South Asians exhibited worse outcomes for an income bracket of $40,227 to $50,333 compared with the lowest income bracket of <$40,227 (HR, 1.46; 95% CI, 1.05–2.03; *P* = 0.025), with no differences observed in education or rural–urban residency. Pacific Islanders had poorer outcomes in rural residency when compared with urban residency (HR, 10.1; 95% CI, 1.07–95.3; *P* = 0.043). For East Asians, patients within the income quartile $50,354 to 63,332 experienced better survival outcomes compared with those within the lowest income quartile of <$40,227 (HR, 0.85; 95% CI, 0.74–0.97; *P* = 0.016).

### Stage and survival

With regard to cancer staging, a predominant portion of the study cohort was classified as AJCC stage IV, accounting for 47.3% of the total cohort. Upon closer examination of the clinical outcomes in relation to AJCC staging, a progressive decline in patient prognosis was observed with advancing stages. Individuals with stage II, stage III, and stage IV disease exhibited progressively higher HRs compared with stage I (stage II HR, 1.75; 95% CI, 1.62–1.90; stage III HR, 2.53; 95% CI, 2.32–2.76; stage IV HR, 4.50; 95% CI, 4.18–4.85; all *P* < 0.001). Southeast Asians were disproportionately represented in this advanced stage, with 50.5% (*n* = 1,359) of their subgroup being stage IV compared with the other races. On multivariable analysis, as the AJCC stage progressed, outcomes worsened.

In the multivariable analysis, East Asians and Pacific Islanders had worse outcomes than South Asians, with no difference in outcomes for Southeast Asians (Table 3). Despite Southeast Asians showing the worst outcomes in univariate analysis, after adjusting for confounders in multivariate analysis, they were not significant compared with South Asians (*P* = 0.11). Therefore, a multivariable analysis setting the Southeast Asian subgroup as the control population was conducted (Table 4). Notably, age and gender emerged as protective factors. Individuals younger than 65 years demonstrated better outcomes compared with their older counterparts ages >65 years (HR, 0.63; 95% CI, 0.55–0.71; *P* < 0.001). Similarly, the female gender was also associated with more favorable outcomes, suggesting a protective effect of being female within this subgroup compared with males (HR, 0.90; 95% CI, 0.81–0.99; *P* = 0.03). These findings suggest that younger age and female gender are protective factors against mortality.

**Table 3:** Multivariable survival analysis for patients diagnosed with PDAC

Characteristic	HR	95% CI	*P* value^a^
Asian group			
South Asian	—	—	
East Asian	1.10	1.02–1.18	0.017
Pacific Islander	1.22	1.09–1.37	<0.001
Southeast Asian	1.07	0.98–1.17	0.11
Age group			
≥65	—	—	
<65	0.6	0.56–0.64	<0.001
Gender			
Male	—	—	
Female	0.99	0.94–1.03	0.6
Insurance status			
Not insured	—	—	
Insurance status unknown	1.03	0.83–1.27	0.8
Medicaid/other government programs	0.92	0.80–1.07	0.3
Medicare	0.95	0.83–1.08	0.4
Private insurance/managed care	0.97	0.85–1.11	0.7
Median household income			
<$40,227	—	—	
$40,227–$50,353	1.01	0.91–1.13	0.8
$50,354–$63,332	0.88	0.80–0.98	0.017
$63,333+	0.93	0.84–1.03	0.2
No high school degree			
<6.3%	—	—	
6.3%–10.8%	1.06	0.99–1.12	0.08
10.9%–17.5%	1.03	0.96–1.11	0.4
17.6%+	0.99	0.91–1.08	0.9
Rural–urban residence			
Urban	—	—	
Metropolitan	0.87	0.75–1.01	0.06
Rural	0.79	0.55–1.13	0.2
Greater circle distance			
Short (<12.5 minutes)	—	—	
Intermediate (12.5–50 minutes)	0.87	0.82–0.92	<0.001
Long (>50 minutes)	0.82	0.73–0.91	<0.001
Facility location			
West South Central	—	—	
East North Central	1.04	0.91–1.20	0.6
East South Central	1.23	0.96–1.57	0.1
Middle Atlantic	0.77	0.69–0.87	<0.001
Mountain	1.18	0.99–1.42	0.07
New England	0.95	0.80–1.13	0.6
Pacific	1.14	1.02–1.27	0.021
South Atlantic	0.95	0.84–1.08	0.5
West North Central	1.13	0.95–1.34	0.2
Facility type			
Community	—	—	
Academic	0.93	0.88–0.97	0.003
Charlson–Deyo score			
0	—	—	
1	1.03	0.98–1.09	0.2
2	1.24	1.12–1.38	<0.001
3+	1.33	1.18–1.51	<0.001
Diagnosis era			
2005–2009	—	—	
2010–2014	0.82	0.77–0.87	<0.001
2015–2019	0.65	0.62–0.69	<0.001
N stage			
N0	—	—	
N1	1.11	1.05–1.18	<0.001
N2/Nx	1.18	1.10–1.26	<0.001
M stage			
M0	—	—	
M1/Mx	1.14	1.00–1.30	0.05
AJCC staging			
Stage I	—	—	
Stage II	1.67	1.52–1.82	<0.001
Stage III	1.74	1.57–1.92	<0.001
Stage IV	2.69	2.32–3.12	<0.001
Surgery (time-dependent)			
No	—		
Yes	0.47	0.44–0.51	<0.001

a *P* values set for significance <0.05.

**Table 4:** Southeast Asian subgroup survival analysis for patients diagnosed with PDAC

Characteristic	HR	95% CI	*P* value^a^
Age group			
≥65	—	—	
<65	0.63	0.55–0.71	<0.001
Gender			
Male	—	—	
Female	0.9	0.81–0.99	0.03
Insurance status			
Not insured	—	—	
Insurance status unknown	1.08	0.65–1.80	0.8
Medicaid/other government programs	0.97	0.71–1.32	0.8
Medicare	1	0.74–1.33	>0.9
Private insurance/managed care	0.99	0.74–1.31	>0.9
Median household income			
<$40,227	—	—	
$40,227–$50,353	1.01	0.81–1.26	>0.9
$50,354–$63,332	0.88	0.72–1.08	0.2
$63,333+	0.89	0.72–1.10	0.3
No high school degree			
<6.3%	—	—	
6.3%–10.8%	0.89	0.76–1.04	0.1
10.9%–17.5%	0.97	0.82–1.14	0.7
17.6%+	0.91	0.76–1.09	0.3
Rural–urban residence			
Urban	—	—	
Metropolitan	0.69	0.50–0.97	0.03
Rural	0.66	0.23–1.88	0.4
Greater circle distance			
Short (<12.5 minutes)	—	—	
Intermediate (12.5–50 minutes)	0.92	0.81–1.04	0.2
Long (>50 minutes)	0.96	0.76–1.21	0.7
Facility location			
West South Central	—	—	
East North Central	0.95	0.69–1.31	0.8
East South Central	0.47	0.22–1.00	0.05
Middle Atlantic	0.78	0.58–1.04	0.09
Mountain	0.96	0.64–1.44	0.8
New England	0.74	0.51–1.08	0.12
Pacific	0.94	0.73–1.21	0.6
South Atlantic	0.83	0.61–1.12	0.2
West North Central	0.94	0.67–1.32	0.7
Facility type			
Community	—	—	
Academic	0.96	0.87–1.06	0.4
Charlson–Deyo score			
0	—	—	
1	1	0.89–1.12	>0.9
2	1.37	1.12–1.68	0.002
3+	1.28	0.97–1.68	0.08
Diagnosis era			
2005–2009	—	—	
2010–2014	0.9	0.79–1.03	0.11
2015–2019	0.72	0.63–0.81	<0.001
N stage			
N0	—	—	
N1	1.12	1.00–1.26	0.06
N2/Nx	1.21	1.05–1.39	0.01
M stage			
M0	—	—	
M1/Mx	1.08	0.82–1.41	0.6
AJCC staging			
Stage I	—	—	
Stage II	1.67	1.37–2.03	<0.001
Stage III	1.76	1.42–2.17	<0.001
Stage IV	2.75	2.02–3.75	<0.001
Surgery (time-dependent)			
No			
Yes	0.48	0.40–0.56	<0.001

a *P* values set for significance <0.05.

## Discussion

In the realm of PDAC research, the classification of patients by broad geographic ancestry such as Asian can obscure significant underlying disparities. This grouping overlooks the considerable genetic, cultural, and social determinants within Asian populations, which may contribute to differential disease prevalence and survival rates (13, 14). Acknowledging this diversity is crucial, as it may uncover unique risk factors and lead to more effective, tailored interventions (15). Therefore, our study attempts to refine this broad categorization by distinguishing among East Asians, Southeast Asians, South Asians, and Pacific Islanders. This detailed stratification aims to illuminate survival outcomes in PDAC with greater precision and cultural sensitivity.

SDOH encompass a broader range of factors beyond just race; they include income, education status, and the availability of community support systems, which are all vital in shaping health-seeking behaviors and healthcare accessibility (16). Research has shown that barriers such as language proficiency and immigration status can hamper healthcare access for Southeast Asians in particular, often resulting in later-stage diagnoses and suboptimal health outcomes (17, 18). Conversely, South Asians, who generally have a higher socioeconomic status and are more integrated into healthcare systems, tend to be diagnosed with more favorable prognostic factors (19, 20).

In our multivariable analyses, a more refined depiction of survival outcomes among Asians emerged. Although bivariate analysis initially suggested that Southeast Asians had the worst OS rates, this association dissipated when controlling for a range of SDOH, clinical, and cancer staging factors. This finding emphasizes the intricate relationship between ethnicity and a host of other variables that collectively shape health outcomes. It is indicative of the fact that when SDOH, such as education level, income, health literacy, and community support, are accounted for, along with clinical presentations and treatment regimens, the survival disadvantage seen in Southeast Asians is not inherently correlated with ethnicity itself.

The potential role of genetic factors in influencing PDAC outcomes is an area still under evaluation. It is possible that certain genetic polymorphisms prevalent within Southeast Asian populations may offer some degree of protection against the progression or development of PDAC (21). Conversely, it is important to acknowledge that certain genetic factors may predispose East Asian and Pacific Islander populations to poorer clinical outcomes in PDAC. Enzymatic functions involved in metabolism, DNA repair pathways that maintain genomic integrity, and gene variations that regulate immune responses may all differ across ethnic lines (22, 23). The observed diversity in genetic makeup can contribute to the differences observed in disease progression and patient outcome. Therefore, it is vital to pursue genome-wide association studies within these communities to identify specific alleles that might confer resilience or risks for developing PDAC (24). Such research could pave the way for precision medicine approaches that take into account the genetic profiles unique to Southeast Asians and other races, thereby optimizing prevention strategies and treatment protocols.

Cultural practices, such as those related to diet, also require a closer examination. Traditional Southeast Asian diets, which are rich in certain vegetables, fruits, and spices known for their anti-inflammatory and potentially anticarcinogenic properties, could incidentally contribute to the observed survival patterns (25). Turmeric, ginger, and other spices commonly used in Southeast Asian cuisine contain bioactive compounds that have been the subject of cancer research (26, 27). The epidemiologic examination into these dietary patterns could reveal associations with PDAC survival rates. Furthermore, cultural perspectives on health maintenance, disease prevention, and treatment adherence are all deeply rooted in cultural traditions and can significantly influence health outcomes (28). Understanding and integrating these cultural nuances into healthcare provision could lead to enhanced patient participation and adherence to treatment protocols.

The structure and impact of community support signify a vital aspect that requires in-depth examination when looking at patient outcomes. This includes the roles of social networks, collective resources, and communal coping mechanisms, leading to resilience, as well as the synergistic effects these elements have on individual and collective well-being. In many Southeast Asian cultures, solid community networks provide a strong support system that can promote health education, facilitate access to healthcare, and enhance the management of illness (29). These communal networks frequently function as channels for the transmission of critical health data and facilitation of medical resources, which can positively influence the clinical outcomes of conditions such as PDAC. The strength and structure of these support systems could be an aspect in the enhanced survival outcomes observed when SDOH are balanced across populations. Public health initiatives in the future should be designed to leverage these community networks, improving the effectiveness of health interventions and guaranteeing cultural alignment and positive reception of the measures.

One limitation of utilizing the NCDB is the possibility of selection bias. The database compiles data exclusively from hospital-based registries, which might not accurately reflect the broader general population. Hospitals that contribute to the NCDB could differ in their patient demographics or available treatment options compared with those that do not participate. Furthermore, the potential for foreign-born populations to be lost to follow-up, along with a lower capture of death reports in cancer surveillance programs, may inflate survival estimates, particularly among majority foreign-born Asians (30). Another limitation of our study is the absence of data on certain Asian nationalities in the NCDB, which restricts the comprehensiveness of our analysis across all Asian populations. Future studies should incorporate a larger range of SDOH factors, utilizing standardized measurement tools to achieve a more thorough understanding of health disparities.

The cumulative evidence from our analysis indicates that ethnicity, while important, represents merely a segment of a more extensive and complicated matrix of health determinants. By advancing our understanding of the multifactorial determinants of health, including the genetic and cultural foundations specific to Southeast Asian populations, we must also continue to address the systemic inequalities that influence these outcomes. A comprehensive and integrative strategy is imperative to eliminate the observed disparities in PDAC survival rates and improve the health trajectories for all individuals affected by this challenging disease.
